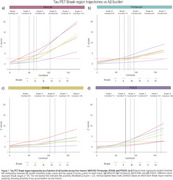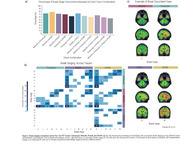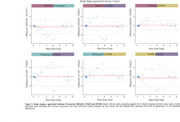# Assessing Braak stage agreement between four Tau PET tracers

**DOI:** 10.1002/alz70862_109849

**Published:** 2025-12-23

**Authors:** Andreia Rocha, Bruna Bellaver, Carolina Soares, Pamela C.L. Ferreira, Emma Ruppert, Marina Scop Madeiros, Guilherme Povala, Livia Amaral, Guilherme Bauer‐Negrini, Firoza Z Lussier, Matheus Scarpatto Rodrigues, Rayan Mroué, Joseph C. Masdeu, Dana L Tudorascu, David N. soleimani‐Meigooni, Juan Fortea, Val J Lowe, Hwamee Oh, Belen Pascual, Brian A. Gordon, Pedro Rosa‐Neto, Suzanne L. Baker, Tharick A Pascoal

**Affiliations:** ^1^ University of Pittsburgh, Pittsburgh, PA USA; ^2^ Houston Methodist Research Institute, Houston, TX USA; ^3^ University of California, San Francisco, San Francisco, CA USA; ^4^ Hospital de la Santa Creu i Sant Pau, Barcelona, Barcelona Spain; ^5^ Department of Radiology, Mayo Clinic, Rochester, MN USA; ^6^ Brown University, Providence, RI USA; ^7^ Washington University in St. Louis, St. Louis, MO USA; ^8^ McGill University, Montreal, QC Canada; ^9^ Lawrence Berkeley National Laboratory, Berkeley, CA USA

## Abstract

**Background:**

The Alzheimer's disease (AD) Braak staging is a key framework for classifying tau pathology progression in AD based on histopathological post‐mortem brain examinations. However, adapting it to PET imaging can be challenging due to differences in tracer uptake patterns and binding properties, which affect sensitivity, specificity, and regional staging. This study compares Braak staging across four tau PET tracers: Flortaucipir, MK6240, PI2620, and RO948.

**Methods:**

We assessed 90 participants across the AD spectrum (46 CU, 31 MCI, 13 dementia; mean age 66.1 ± 7.8) using Aβ PET and four tau PET tracers: (Flortaucipir, MK6240, PI2620, and RO948). Braak positivity was defined based on Aβ− CU individuals (mean +2.5 SD, SUVR). To evaluate systematic bias and agreement between tracers, we computed pairwise differences at corresponding Braak stage estimates and applied the Bland‐Altman method to assess mean bias and limits of agreement. Additionally, Tau PET Braak region trajectories were modeled as functions of Aβ burden (Centiloid scale) using the Lowess method.

**Results:**

Braak stage trajectories as a function of Aβ differ depending on the tracer and the sequential order of abnormality is highly variable. For instance, while for MK6240, RO948 and PI2620, Braak I is the first region to became abnormal, for Flortaucipir the earliest region to became abnormal is Braak IV (Figure 1). This variable pattern of abnormality impacts in the concordance of Braak staging between tracers with the highest Braak staging agreement resulting in concordance levels of approximately 70%. The highest levels of agreement between tracers usually happen at Braak 0 or Braak IV‐V, with intermediate stages showing very low concordance (Figure 2). The Bland‐Altman analysis identified wide limits of agreement, suggesting high variability and high tracer‐specific differences (Figure 3). On the other hand, it also identified that mean differences between tracers were small, indicating minimal systematic bias.

**Conclusion:**

These preliminary findings reveal discrepancies in Braak staging when comparing Flortaucipir, MK6240, PI2620 and RO948. These findings suggest that while the tracers provide comparable stages on average, they may not be fully interchangeable in individual cases.